# Supplementary material for: Loss of Nat4 and its associated histone H4 N‐terminal acetylation mediates calorie restriction‐induced longevity
Source: EMBO Rep. 2016 Oct 31;17(12):1829–43. doi: 10.15252/embr.201642540 (PMC5167350; doi:10.15252/embr.201642540)
Supplement: Supplementary file 1 — Expanded View Figures PDF [file EMBR-17-1829-s001.pdf]

Expanded View Figures

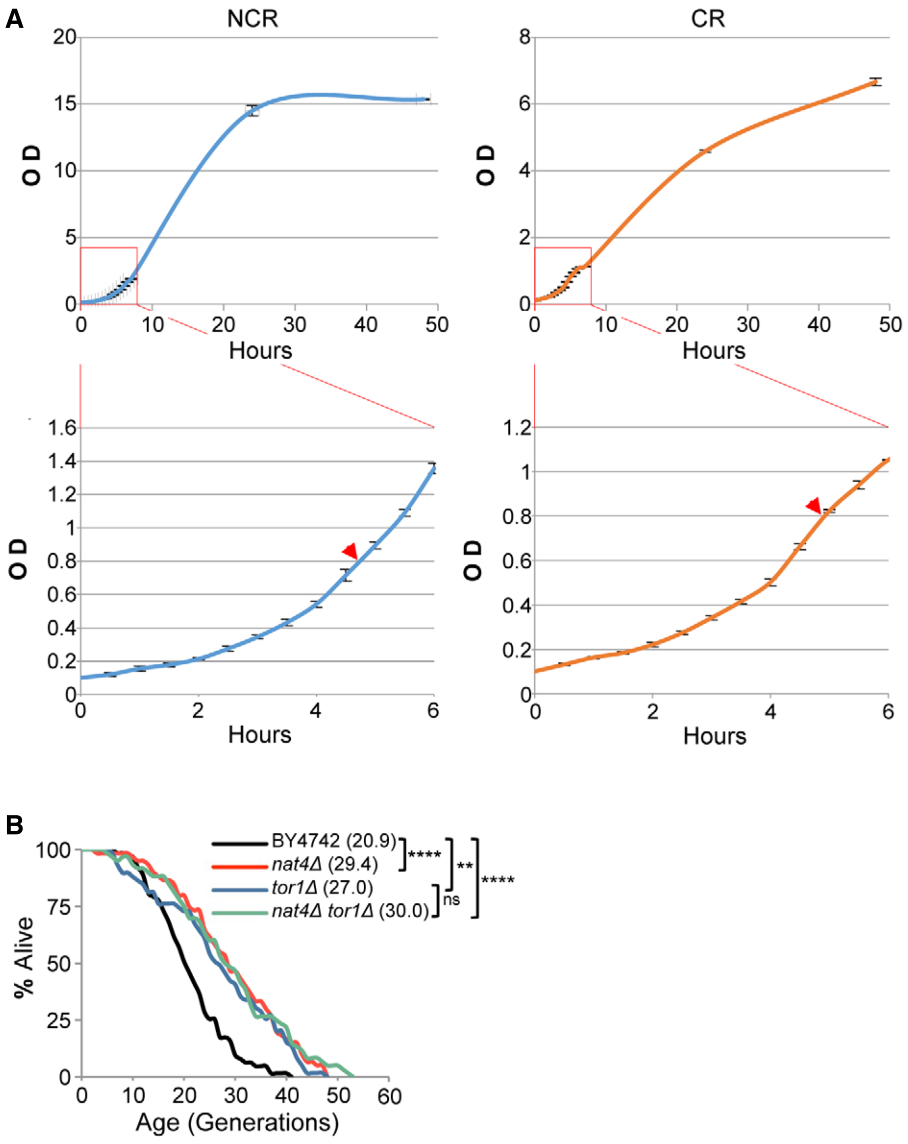

Figure EV1. *nat4*Δ-induced longevity is epistatic to *TOR1* deletion.

- A Growth curves of the BY4741 strain cultured at 30°C in rich YPD medium containing 2% (left) or 0.1% (right) glucose. Cells were harvested for downstream applications at O.D. 0.8 (indicated by an arrowhead in the bottom panels) prior to glucose exhaustion and entry to stationary phase.
- B Replicative lifespan (RLS) for BY4742 wild-type, *nat4*Δ, *tor1*Δ, and double-mutant strains. Values in parentheses indicate mean lifespan. Statistical significance was determined by one-way ANOVA test: $^{**}P \leq 0.01$; $^{****}P \leq 0.0001$; ns = non-significant.

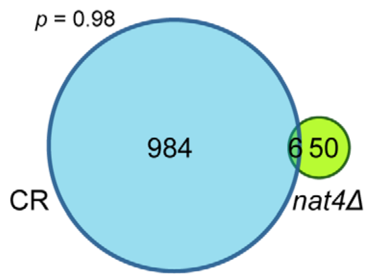

Figure EV2. Overlap of downregulated genes in *nat4*Δ and CR.
Venn diagram showing the overlap (six genes) between downregulated genes in *nat4*Δ cells (56) and wild-type cells grown in calorie restriction conditions (990). Significance of overlap between the two sets is shown as $P = 0.98$ (calculated by hypergeometric test [phyper]).

 © 2016 The Authors

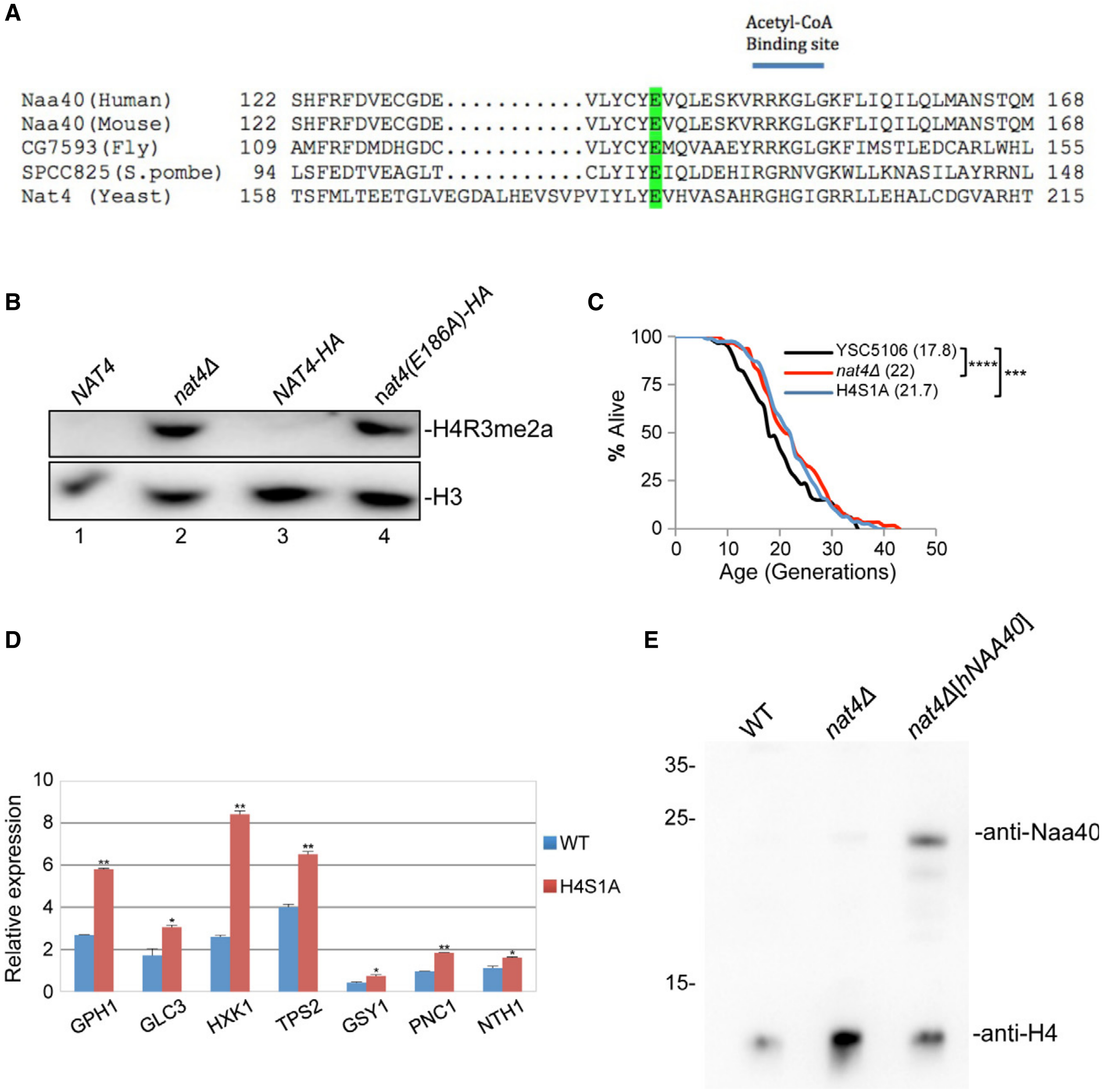

Figure EV3. ***nat4Δ*-induced longevity is mediated through loss of H4 N-terminal acetylation.**

A Sequence alignment showing the conservation of the *NAT4* catalytic motif in five different species. The mutated residue is highlighted in green.

B Whole-cell extracts from strains containing wild-type Nat4 (lanes 1 and 3) a deletion of the *NAT4* gene (lane 2) and Nat4 catalytic mutant (lane 4) were analyzed by Western blotting using an antibody against H4R3me2a. The wild-type in lane 3 and the E186A catalytic mutant (lane 4) have a C-terminal hemagglutinin (HA) tag. Equal loading was monitored using an H3 antibody.

C RLS analysis for YSC5106 wild-type, *nat4*Δ, and H4S1A strains. Values in parentheses indicate mean lifespan. Statistical significance was determined by one-way ANOVA test: ***$P \leq 0.001$; ****$P \leq 0.0001$.

D Gene expression analysis of the indicated stress-induced genes in YSC5106 wild-type and H4S1A strains. Expression levels were normalized to *RPP0* whose expression remains unchanged. Error bars, SEM of three independent experiments. Statistical significance was determined by unpaired two-tailed Student's *t*-test: *$P \leq 0.05$ and **$P \leq 0.01$ compared to wild-type values.

E Whole yeast cell extracts prepared from BY4742 WT(+pBEVY-U), *nat4*Δ(+pBEVY-U), and *nat4*Δ[pBEVY-U-*hNAA40*] strains were analyzed by Western blotting using the indicated antibodies. Loading was monitored with an antibody against histone H4.

© 2016 The Authors

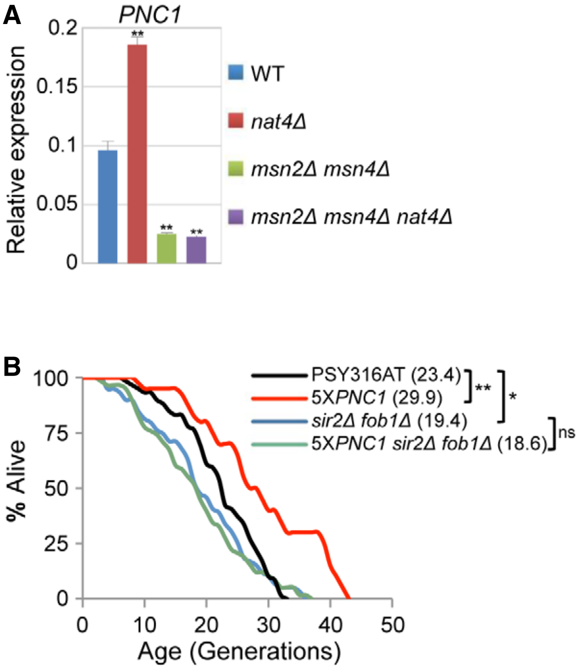

Figure EV4. ***PNC1* overexpression is unable to extend lifespan in cells lacking Sir2 and Fob1.**

A *PNC1* mRNA levels analyzed by qRT–PCR using total RNA extracted from BY4741 wild-type, *nat4Δ* single-, *msn2Δ msn4Δ* double-, and *msn2Δ msn4Δ nat4Δ* triple-mutant strains in 2% glucose. Expression levels were normalized to *ACT1*, whose expression remains unchanged. Error bars, SEM of three independent experiments. Statistical significance was determined by unpaired two-tailed Student's *t*-test: $^{**}P \leq 0.01$ compared to wild-type values.

B Replicative lifespan (RLS) for PSY316AT wild-type, 5x*PNC1*, *sir2Δ fob1Δ*, and 5x*PNC1 sir2Δ fob1Δ* mutant strains. Values in parentheses indicate mean lifespan. Statistical significance was determined by one-way ANOVA test: $^{*}P \leq 0.05$; $^{**}P \leq 0.01$; ns = non-significant.

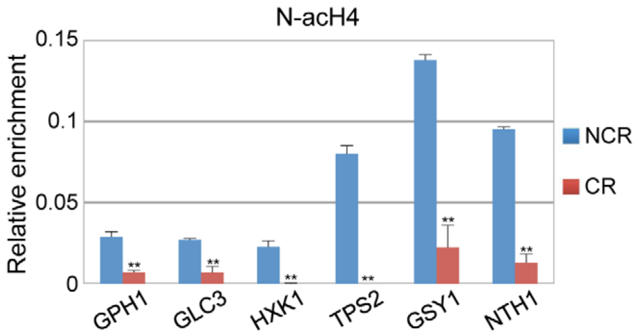

Figure EV5. CR reduces the N-acH4 levels at the promoter of several stress-response genes.

ChIP analysis performed in BY4741 wild-type strain grown under NCR (2% glucose) and CR (0.1% glucose) conditions. Chromatin was immunoprecipitated using antibodies against N-acH4 and histone H4 and analyzed by qRT–PCR with primers corresponding to the indicated genes. The enrichment from the antibody was normalized to histone H4. Error bars, SEM of three independent experiments. Statistical significance was determined by unpaired two-tailed Student's *t*-test: $^{**}P \leq 0.01$ compared CR to NCR values.

 © 2016 The Authors
