# Supplementary material for: Loss of Nat4 and its associated histone H4 N‐terminal acetylation mediates calorie restriction‐induced longevity
Source: EMBO Rep. 2016 Oct 31;17(12):1829–43. doi: 10.15252/embr.201642540 (PMC5167350; doi:10.15252/embr.201642540)
Supplement: Supplementary file 2 — Table EV1 [file EMBR-17-1829-s002.docx]

**Table EV1**: List of upregulated genes in *nat4Δ* [abs(logFC) >= 1 and FDR <= 0.0001]

| **Systematic name** | **Gene name** | **Fold increase (log2)** |
| --- | --- | --- |
| *YMR316C-A* | *YMR316C-A* | 11,07649535 |
| *YFL062W* | *COS4* | 8,984687038 |
| *YOR178C* | *GAC1* | 5,345700228 |
| *YNL194C* | *YNL194C* | 4,964345855 |
| *YPR160W* | *GPH1* | 4,924315444 |
| *YFR015C* | *GSY1* | 4,73739335 |
| *YFR053C* | *HXK1* | 4,623250909 |
| *YNR034W-A* | *YNR034W-A* | 4,40140966 |
| *YDR343C* | *HXT6* | 4,386026627 |
| *YEL011W* | *GLC3* | 4,301204678 |
| *YER067W* | *RGI1* | 4,270075904 |
| *YLR327C* | *TMA10* | 4,22738026 |
| *YBR147W* | *RTC2* | 4,043516068 |
| *YDR342C* | *HXT7* | 3,726482024 |
| *YLR377C* | *FBP1* | 3,696865544 |
| *YMR206W* | *YMR206W* | 3,676166551 |
| *YOL052C-A* | *DDR2* | 3,562564452 |
| *YJL052W* | *TDH1* | 3,50985567 |
| *YHR092C* | *HXT4* | 3,465944624 |
| *YMR081C* | *ISF1* | 3,253686294 |
| *YGR138C* | *TPO2* | 3,117616694 |
| *YMR105C* | *PGM2* | 3,020634985 |
| *YLR258W* | *GSY2* | 2,833571337 |
| *YML100W* | *TSL1* | 2,732598053 |
| *YDL169C* | *UGX2* | 2,664444066 |
| *YLR149C* | *YLR149C* | 2,620722689 |
| *YCR021C* | *HSP30* | 2,591791687 |
| *YER037W* | *PHM8* | 2,478030942 |
| *YGR008C* | *STF2* | 2,473577317 |
| *YOR134W* | *BAG7* | 2,451652415 |
| *YFR017C* | *IGD1* | 2,450319261 |
| *YGR088W* | *CTT1* | 2,306550474 |
| *YNL202W* | *SPS19* | 2,284370239 |
| *YNR002C* | *ATO2* | 2,250100337 |
| *YCL040W* | *GLK1* | 2,234208323 |
| *YMR105W-A* | *YMR105W-A* | 2,222085961 |
| *YOR049C* | *RSB1* | 2,207872102 |
| *YDR034W-B* | *YDR034W-B* | 2,151431407 |
| *YPL230W* | *USV1* | 2,100081335 |
| *YGR052W* | *FMP48* | 2,069733589 |
| *YKL163W* | *PIR3* | 2,059161581 |
| *YDR516C* | *EMI2* | 2,051342715 |
| *YHL021C* | *AIM17* | 2,03904017 |
| *YMR280C* | *CAT8* | 2,027553773 |
| *YMR250W* | *GAD1* | 2,022106373 |
| *YPL186C* | *UIP4* | 2,015978623 |
| *YFL052W* | *YFL052W* | 1,955274864 |
| *YPL054W* | *LEE1* | 1,926871149 |
| *YIL101C* | *XBP1* | 1,916442949 |
| *YGR248W* | *SOL4* | 1,869828256 |
| *YJL161W* | *FMP33* | 1,816447159 |
| *YDL048C* | *STP4* | 1,78577496 |
| *YDL079C* | *MRK1* | 1,772919784 |
| *YGR023W* | *MTL1* | 1,769954574 |
| *YBR093C* | *PHO5* | 1,768009433 |
| *YJR115W* | *YJR115W* | 1,752615548 |
| *YDR074W* | *TPS2* | 1,746362509 |
| *YIL169C* | *YIL169C* | 1,717605256 |
| *YER053C* | *PIC2* | 1,709945371 |
| *YOR347C* | *PYK2* | 1,701133875 |
| *YMR104C* | *YPK2* | 1,700486254 |
| **Systematic name** | **Gene name** | **Fold increase (log2)** |
| *YOL155C* | *HPF1* | 1,672894948 |
| *YAL061W* | *BDH2* | 1,667261831 |
| *YPR194C* | *OPT2* | 1,64979729 |
| *YDL204W* | *RTN2* | 1,62843353 |
| *YDR247W* | *VHS1* | 1,623407259 |
| *YBR294W* | *SUL1* | 1,620049947 |
| *YHR087W* | *RTC3* | 1,620049947 |
| *YOR273C* | *TPO4* | 1,608129923 |
| *YJR008W* | *YJR008W* | 1,589097018 |
| *YOR173W* | *DCS2* | 1,576928678 |
| *YBR230W-A* | *YBR230W-A* | 1,558649402 |
| *YLR178C* | *TFS1* | 1,553412754 |
| *YKL044W* | *YKL044W* | 1,550787284 |
| *YBR183W* | *YPC1* | 1,510261801 |
| *YNR014W* | *YNR014W* | 1,487535575 |
| *YJL133C-A* | *YJL133C-A* | 1,480397956 |
| *YPL014W* | *YPL014W* | 1,463949942 |
| *YJL144W* | *YJL144W* | 1,452939961 |
| *YBR214W* | *SDS24* | 1,450201099 |
| *YLR142W* | *PUT1* | 1,441942889 |
| *YDL181W* | *INH1* | 1,429916088 |
| *YNL274C* | *GOR1* | 1,424578825 |
| *YGR254W* | *ENO1* | 1,423318929 |
| *YDR277C* | *MTH1* | 1,416730459 |
| *YGL037C* | *PNC1* | 1,412626984 |
| *YKR058W* | *GLG1* | 1,405488273 |
| *YNL144C* | *YNL144C* | 1,395696009 |
| *YMR136W* | *GAT2* | 1,385749515 |
| *YBR054W* | *YRO2* | 1,365189472 |
| *YPL165C* | *SET6* | 1,364669113 |
| *YGR249W* | *MGA1* | 1,357599983 |
| *YMR084W* | *YMR084W* | 1,357015541 |
| *YDR171W* | *HSP42* | 1,345458509 |
| *YML120C* | *NDI1* | 1,329989058 |
| *YCL025C* | *AGP1* | 1,319019028 |
| *YNL160W* | *YGP1* | 1,290131863 |
| *YCR024C-B* | *YCR024C-B* | 1,281644414 |
| *YIR039C* | *YPS6* | 1,276962289 |
| *YLL039C* | *UBI4* | 1,270303908 |
| *YEL017C-A* | *PMP2* | 1,262688158 |
| *YPL250C* | *ICY2* | 1,260053811 |
| *YPL087W* | *YDC1* | 1,238027505 |
| *YKL062W* | *MSN4* | 1,228335462 |
| *YGR289C* | *MAL11* | 1,225431989 |
| *YLR177W* | *YLR177W* | 1,224688497 |
| *YBR033W* | *EDS1* | 1,214996536 |
| *YBR132C* | *AGP2* | 1,212067205 |
| *YKR091W* | *SRL3* | 1,204005431 |
| *YPR030W* | *CSR2* | 1,203695519 |
| *YGR110W* | *CLD1* | 1,185249193 |
| *YPR157W* | *TDA6* | 1,176998071 |
| *YDR216W* | *ADR1* | 1,175757223 |
| *YKL137W* | *CMC1* | 1,172275661 |
| *YJL164C* | *TPK1* | 1,171845583 |
| *YOL048C* | *RRT8* | 1,171565964 |
| *YDL244W* | *THI13* | 1,164370463 |
| *YER150W* | *SPI1* | 1,160863099 |
| *YIL113W* | *SDP1* | 1,154638925 |
| *YKL148C* | *SDH1* | 1,153976361 |
| *YDR380W* | *ARO10* | 1,150086237 |
| *YBR296C* | *PHO89* | 1,124133871 |
| *YDR085C* | *AFR1* | 1,118855804 |

| **Systematic name** | **Gene name** | **Fold increase (log2)** |
| --- | --- | --- |
| *YML128C* | *MSC1* | 1,117497059 |
| *YPR184W* | *GDB1* | 1,116082989 |
| *YNR001C* | *CIT1* | 1,111483716 |
| *YML118W* | *NGL3* | 1,097256267 |
| *YJL141C* | *YAK1* | 1,094768886 |
| *YOR065W* | *CYT1* | 1,086110143 |
| *YOR289W* | *YOR289W* | 1,083480723 |
| *YER079W* | *YER079W* | 1,06127697 |
| *YLR267W* | *BOP2* | 1,053261375 |
| *YFL030W* | *AGX1* | 1,048893245 |
| *YCL042W* | *YCL042W* | 1,043156876 |
| *YCR091W* | *KIN82* | 1,033816909 |
| *YPR036W-A* | *YPR036W-A* | 1,030643401 |
| *YKR046C* | *PET10* | 1,018940023 |
| *YLL041C* | *SDH2* | 1,006587123 |
