# Supplementary material for: Loss of Nat4 and its associated histone H4 N‐terminal acetylation mediates calorie restriction‐induced longevity
Source: EMBO Rep. 2016 Oct 31;17(12):1829–43. doi: 10.15252/embr.201642540 (PMC5167350; doi:10.15252/embr.201642540)
Supplement: Supplementary file 3 — Table EV2 [file EMBR-17-1829-s003.docx]

**Table EV2:** List of downregulated genes in *nat4Δ* [abs(logFC) >= 1 and FDR <= 0.0001]

| **Systematic name** | **Gene name** | **Fold decrease (log2)** |
| --- | --- | --- |
| *YMR069W* | *NAT4* | -10,39129272 |
| *YJR150C* | *DAN1* | -2,642984459 |
| *YLR151C* | *PCD1* | -2,642984459 |
| *YHR219W* | *YHR219W* | -2,379950053 |
| *YDR366C* | *YDR366C* | -1,894523226 |
| *YER011W* | *TIR1* | -1,890462121 |
| *YJR047C* | *ANB1* | -1,852598276 |
| *YIR005W* | *IST3* | -1,758461677 |
| *YDR106W* | *ARP10* | -1,602342475 |
| *YJR112W* | *NNF1* | -1,558226841 |
| *YDL209C* | *CWC2* | -1,514578727 |
| *YLR466W* | *YRF1-4* | -1,473059458 |
| *YPL168W* | *YPL168W* | -1,431065369 |
| *YNL304W* | *YPT11* | -1,409096399 |
| *YER137C* | *YER137C* | -1,347528576 |
| *YDL200C* | *MGT1* | -1,347528576 |
| *YJL205C* | *NCE101* | -1,305949472 |
| *YKR104W* | *YKR104W* | -1,28041438 |
| *YBR257W* | *POP4* | -1,257959529 |
| *YMR117C* | *SPC24* | -1,224671828 |
| *YMR179W* | *SPT21* | -1,224671828 |
| *YER127W* | *LCP5* | -1,216567898 |
| *YHR060W* | *VMA22* | -1,198884494 |
| *YIL144W* | *TID3* | -1,186755273 |
| *YIR015W* | *RPR2* | -1,179037359 |
| *YPR101W* | *SNT309* | -1,176963031 |
| *YGR188C* | *BUB1* | -1,176416659 |
| *YNL289W* | *PCL1* | -1,174179853 |
| *YOR073W* | *SGO1* | -1,172279138 |
| *YMR268C* | *PRP24* | -1,160870366 |
| *YJL162C* | *JJJ2* | -1,154484798 |
| *YGR109C* | *CLB6* | -1,14478327 |
| *YAR068W* | *YAR068W* | -1,143203858 |
| *YIL029C* | *YIL029C* | -1,138192307 |
| *YBR152W* | *SPP381* | -1,126501696 |
| *YDL003W* | *MCD1* | -1,117701643 |
| *YKR029C* | *SET3* | -1,104457531 |
| *YGR079W* | *YGR079W* | -1,100310643 |
| *YLR103C* | *CDC45* | -1,061024447 |
| *YBL010C* | *YBL010C* | -1,058021958 |
| *YJR152W* | *DAL5* | -1,058021958 |
| *YNL110C* | *NOP15* | -1,049459945 |
| *YHR216W* | *IMD2* | -1,031495664 |
| *YPL174C* | *NIP100* | -1,026771024 |
| *YDR528W* | *HLR1* | -1,026486385 |
| *YPL024W* | *RMI1* | -1,02535577 |
| *YMR198W* | *CIK1* | -1,025232023 |
| *YOR295W* | *UAF30* | -1,02325654 |
| *YPL216W* | *YPL216W* | -1,020219926 |
| *YIL019W* | *FAF1* | -1,018312249 |
| *YCL058C* | *ADF1* | -1,009112358 |
| *YCL058W-A* | *ADF1* | -1,009112358 |
| *YJR149W* | *YJR149W* | -1,007197699 |
| *YPL051W* | *ARL3* | -1,00703803 |
| *YNL188W* | *KAR1* | -1,006491658 |
| *YPL146C* | *NOP53* | -1,006437461 |
| *YEL003W* | *GIM4* | -1,00143843 |
