# Supplementary material for: Loss of Nat4 and its associated histone H4 N‐terminal acetylation mediates calorie restriction‐induced longevity
Source: EMBO Rep. 2016 Oct 31;17(12):1829–43. doi: 10.15252/embr.201642540 (PMC5167350; doi:10.15252/embr.201642540)
Supplement: Supplementary file 4 — Table EV3 [file EMBR-17-1829-s004.docx]

**Table EV3**: Common deregulated genes in *nat4Δ* and CR [abs(logFC) >= 1 and FDR <= 0.0001]

| **Systematic name** | **Gene name** | **Up/Downregulated** |
| --- | --- | --- |
| *YDR216W* | *ADR1* | UP |
| *YDR085C* | *AFR1* | UP |
| *YBR132C* | *AGP2* | UP |
| *YFL030W* | *AGX1* | UP |
| *YJR047C* | *ANB1* | DOWN |
| *YNR002C* | *ATO2* | UP |
| *YOR134W* | *BAG7* | UP |
| *YAL061W* | *BDH2* | UP |
| *YLR267W* | *BOP2* | UP |
| *YMR280C* | *CAT8* | UP |
| *YNR001C* | *CIT1* | UP |
| *YKL137W* | *CMC1* | UP |
| *YPR030W* | *CSR2* | UP |
| *YGR088W* | *CTT1* | UP |
| *YOR065W* | *CYT1* | UP |
| *YOR173W* | *DCS2* | UP |
| *YOL052C-A* | *DDR2* | UP |
| *YBR033W* | *EDS1* | UP |
| *YDR516C* | *EMI2* | UP |
| *YLR377C* | *FBP1* | UP |
| *YJL161W* | *FMP33* | UP |
| *YOR178C* | *GAC1* | UP |
| *YMR250W* | *GAD1* | UP |
| *YPR184W* | *GDB1* | UP |
| *YEL011W* | *GLC3* | UP |
| *YKR058W* | *GLG1* | UP |
| *YCL040W* | *GLK1* | UP |
| *YOR348C* | *GOR1* | UP |
| *YPR160W* | *GPH1* | UP |
| *YFR015C* | *GSY1* | UP |
| *YLR258W* | *GSY2* | UP |
| *YOL155C* | *HPF1* | UP |
| *YDR171W* | *HSP42* | UP |
| *YFR053C* | *HXK1* | UP |
| *YDR343C* | *HXT6* | UP |
| *YDR342C* | *HXT7* | UP |
| *YHR216W* | *IMD2* | DOWN |
| *YDL181W* | *INH1* | UP |
| *YMR081C* | *ISF1* | UP |
| *YCR091W* | *KIN82* | UP |
| *YER127W* | *LCP5* | DOWN |
| *YPL054W* | *LEE1* | UP |
| *YGR289C* | *MAL11* | UP |
| *YDL079C* | *MRK1* | UP |
| *YML128C* | *MSC1* | UP |
| *YDR277C* | *MTH1* | UP |
| *YMR069W* | *NAT4* | DOWN |
| *YML120C* | *NDI1* | UP |
| *YML118W* | *NGL3* | UP |
| *YKR046C* | *PET10* | UP |
| *YMR105C* | *PGM2* | UP |
| *YGL037C* | *PNC1* | UP |
| *YLR142W* | *PUT1* | UP |
| *YOR347C* | *PYK2* | UP |
| *YDL204W* | *RTN2* | UP |
| *YKL148C* | *SDH1* | UP |
| *YBR214W* | *SDS24* | UP |
| *YGR248W* | *SOL4* | UP |
| *YER150W* | *SPI1* | UP |
| *YNL202W* | *SPS19* | UP |
| *YGR008C* | *STF2* | UP |
| **Systematic name** | **Gene name** | **Up/Downregulated** |
| *YLR178C* | *TFS1* | UP |
| *YER011W* | *TIR1* | DOWN |
| *YLR327C* | *TMA10* | UP |
| *YJL164C* | *TPK1* | UP |
| *YML100W* | *TSL1* | UP |
| *YLL039C* | *UBI4* | UP |
| *YDL169C* | *UGX2* | UP |
| *YPL186C* | *UIP4* | UP |
| *YIL101C* | *XBP1* | UP |
| *YBR230W-A* | *YBR230W-A* | UP |
| *YPL087W* | *YDC1* | UP |
| *YER079W* | *YER079W* | UP |
| *YFL052W* | *YFL052W* | UP |
| *YNL160W* | *YGP1* | UP |
| *YGR079W* | *YGR079W* | DOWN |
| *YJR008W* | *YJR008W* | UP |
| *YKL044W* | *YKL044W* | UP |
| *YLR149C* | *YLR149C* | UP |
| *YLR177W* | *YLR177W* | UP |
| *YMR084W* | *YMR084W* | UP |
| *YMR206W* | *YMR206W* | UP |
| *YNL144C* | *YNL144C* | UP |
| *YNL194C* | *YNL194C* | UP |
| *YNR034W-A* | *YNR034W-A* | UP |
| *YOR289W* | *YOR289W* | UP |
| *YBR183W* | *YPC1* | UP |
| *YMR104C* | *YPK2* | UP |
| *YIR039C* | *YPS6* | UP |
