# Supplementary material for: Loss of Nat4 and its associated histone H4 N‐terminal acetylation mediates calorie restriction‐induced longevity
Source: EMBO Rep. 2016 Oct 31;17(12):1829–43. doi: 10.15252/embr.201642540 (PMC5167350; doi:10.15252/embr.201642540)
Supplement: Supplementary file 5 — Table EV4 [file EMBR-17-1829-s005.docx]

**Table EV4**: List of yeast strains

| **Name** | **Genotype** | **Reference** |
| --- | --- | --- |
| BY4741 | *MAT****a****, ura3Δ0, leu2Δ0, his3Δ1, met15Δ0* | Euroscarf |
| Y06202 | Same as BY4741, with *nat4::KanMX4* | Euroscarf |
| AK634 | Same as BY4741, with *Pste5-NAT4::NatMX* | This work |
| BY4742 | *MAT****α****, ura3Δ0, leu2Δ0, his3Δ1, lys2Δ0* | Euroscarf |
| DH461 | Same as BY4742, with *tor1::URA3* | Steffen, K.K. *et al*. (2008), *Cell* **133**, 292 |
| AK509 | Same as BY4742, with *nat4::NatMX4* | This work |
| AK511 | Same as BY4742, with *tor1::URA3, nat4::KanMX4* | This work |
| SF10 | BJ5459, *MAT****a*** *ura3-52, trp1, lys2-801, leu2Δ1, his3Δ200, pep4::HIS3, prb1Δ1.6R can1* | Huang, J. and Moazed, D. (2003), *Genes Dev*, **17**, 2162 |
| DMY1690 | SF10 with *NET1-TAP::K.l-TRP1* | Huang, J. and Moazed, D. (2003), *Genes Dev*, **17**, 2162 |
| AK568 | Same as SF10, with *nat4::KanMX* | This work |
| AK569 | Same as DMY1690, with *nat4::KanMX* | This work |
| AK563 | Same as BY4741 except *nat4::nat4E186A-HA-KanMX4* | This work |
| YSC5106 | *MAT****a****, his3Δ200, leu2Δ0, lys2Δ0, trp1Δ63, ura3Δ0, met15Δ0, can1::MFA1pr-HIS3, hht1-hhf1::NatMX4, hht2-hhf2::*[*HHTS-HHFS*]**-URA3* | Open biosystems |
| AK315 | Same as YSC5106, with *nat4::KanMX4* | Schiza, V. *et al.* (2013), *PLoS Genetics*, **9**:e1003805 |
| YSC5106 H4S1D | Same as YSC5106, except *hht2-hhf2::*[*HHTS-HHFS S1D*]*-*URA3* | Open biosystems |
| YSC5106 H4S1A | Same as YSC5106, except *hht2-hhf2::*[*HHTS-HHFS S1A*]*-*URA3* | Open biosystems |
| YSC5106 H4R3K | Same as YSC5106, except *hht2-hhf2::*[*HHTS-HHFS R3K*]*-*URA3* | Open biosystems |
| AK318 | Same as H4R3K, with *nat4:NatMX4* | Schiza, V. *et al.* (2013), *PLoS Genetics*, **9**:e1003805 |
| Y10000 pBEVY-U | *MAT****α****, ura3Δ0, leu2Δ0, his3Δ1, lys2Δ0* + pBEVY-U | Hole, K. *et al*. (2011), *PLoS One* **6**:e24713 |
| Y16202 pBEVY-U | Same as Y10000 pBEVY-U, with *nat4::KanMX4* | Hole, K. *et al*. (2011), *PLoS One* **6**:e24713 |
| Y16202 pBEVY-U-h*NAA40* | Same as Y16202 pBEVY-U, except pBEVY-U-h*NAA40* | Hole, K. *et al*. (2011), *PLoS One* **6**:e24713 |
| Y04405 | Same as BY4741, with *pnc1::KanMX4* | Euroscarf |
| AK490 | Same as BY4741, with *pnc1::KanMX4*, *nat4::NatMX4* | This work |
| JK9-3D***α*** | *MATα leu2-3,112*, *ura3-52*, *rme1*, *trp1*, *his4* | Miranda, T.B. *et al.* (2006), *Biochem. J.* **395**, 563 |
| AK365 | Same as JK9-3D**α**, with *nat4:NatMX4* | This work |
| AK502 | Same as BY4741, with *fob1::KanMX4*, *sir2::NatMX4* | This work |
| AK503 | Same as BY4741, with *fob1::URA3*, *nat4::KanMX4*, *sir2::NatMX4* | This work |
| PSY316AT | *MATα*, *ura3-53*, *leu2-3.112*, *his3-Δ200*, *ade2-101*, *can1-100*, *ADE2- TEL V-R* | Mills, K.D. *et al*. (1999), *Cell*, 97, 609 |
| YDS1847 | Same as PSY316AT with 5x*PNC1-URA3* | Anderson, R.M. *et al.* (2003), *Nature*, **423**, 181 |
| AK640 | Same as YDS1847, with *fob1::KanMX4*, *sir2::HIS3* | This work |
| AK641 | Same as YDS1845, with *fob1::KanMX4*, *sir2::HIS3* | This work |
| *msn2Δ/msn4Δ* | Same as BY4741, with *msn2::NatMX4* *msn4::KanMX4* | Dolz-Edo, L. *et al*. (2013), *MCB,* **33**, 636 |
| AK631 | Same as BY4741, with *nat4::BleMX4* | This work |
| AK633 | Same as BY4741, with *msn2::NatMX4*, *msn4::KanMX4*, *nat4::BleMX4* | This work |
