# Supplementary material for: Loss of Nat4 and its associated histone H4 N‐terminal acetylation mediates calorie restriction‐induced longevity
Source: EMBO Rep. 2016 Oct 31;17(12):1829–43. doi: 10.15252/embr.201642540 (PMC5167350; doi:10.15252/embr.201642540)
Supplement: Supplementary file 6 — Table EV5 [file EMBR-17-1829-s006.docx]

**Table EV5**: **Primers used in this work**

| **Primers** | **Forward (5’- 3’)** | **Reverse (5’- 3’)** |
| --- | --- | --- |
| **ChIP assays** | | |
| ***1*** | TGCAAAGATGGGTTGAAAGA | GCTCCATGAAGCAAACTGTC |
| ***2*** | AACAGTGAACAGTGGGGACA | TGCCATCATCATTCCCTAGA |
| ***3*** | CGGTATGCGGAGTTGTAAGA | TACACCCTCGTTTAGTTGCTTCT |
| ***4 (RDN5)*** | TGGTAAGAGCCTGACCGAGT | GATTGCAGCACCTGAGTTTC |
| ***5*** | CATATATTTCTTGTGTGAGAAAGG | GGGTAACCCAGTTCCTCACTA |
| ***6*** | GTTAAGGCAGAGCGACAGAGA | CCTCCCAACTACTTTTCCTCACA |
| ***7 (RDN18)*** | CGTGTACTGGATTTCCAACG | CAAGAGCCACAAGGACTCAA |
| ***8 (RDN58)*** | AACGGATCTCTTGGTTCTCG | TGTGCGTTCAAAGATTCGAT |
| ***9 (RDN25)*** | CGGCGGGAGTAACTATGACT | CCATTCATGCGCGTCACTAA |
| ***10*** | CGACTTAGATGTACAACGGGG | CTCTCTAAACTAGGCCCCGG |
| ***ETS1*** | CAAGAGGGAATAGGTGGGAAAA | GACAAGCATATGACTACTGGCA |
| ***PNC1-P*** | CCGTTTGGCCCACTATGTAT | TCACCTATTGCATCCAACCA |
| ***PNC1-S*** | TTGTGGTCACCAGAGATTGG | CTGGCCTTGGAGAGTGGTAG |
| ***PNC1-E*** | GACCACTGTCCTGCTGGATT | TTGTGGGCCTTCAACTCTTC |
| ***TAF10*** | CCTATCATTCCCGATGCAGT | CCTTGCAATAGCTGCCTAGC |
| ***GPH1-P*** | ACGCCTTCCCCAATTACC | CGTCGGTGTTCCTTCCTTAT |
| ***GLC3-P*** | TCTTGTTCCTGAACGGTGAA | AGGGGTATTGCGGAAGAGAT |
| ***HXK1-P*** | TGGTTGCAAAAACCAACAAA | TCCTCTCAACGGTCCTAAGGT |
| ***TPS2-P*** | CATACAGGGAAATCGGCAGT | GGCGTATTGTCCCAAAGAAA |
| ***GSY1-P*** | CGCCGTAAACGGAATCTTT | GTTCTGTGGCAGGAAATGGT |
| ***NTH1-P*** | GCCTGAAAAGATCGCAAAAC | GGAAAACGATAGGGGGAAAA |
| **Gene expression analysis** | | |
| ***RPP0*** | AACGGTCAAGTGTTCCCATC | AGCGGAAACGAAGTGAGAAA |
| ***TAF10*** | CCTATCATTCCCGATGCAGT | CCTTGCAATAGCTGCCTAGC |
| ***ACT1*** | AGATTCAGAGCCCCAGAAGC | TACCGGCAGATTCCAAACCC |
| ***GPH1*** | TACCACGGCGATTATTACCTG | TTGATTGTGGAACTCCTGGTC |
| ***GLC3*** | TGCTTCTAAAAACGTCGAGGA | CACCCCTACCGGAGCTTATAG |
| ***HXK1*** | GCCGACTCTTTGAAGGACTTT | GGTGTCCTTGGTGTTTAGCA |
| ***TPS2*** | ATCCTGTCACTGTGGGATCTG | TTGCTGAGGATCGGTTAAATG |
| ***GSY1*** | AACAAACGTTTCTGGGTTCG | TGCTCGACAGATTCATCAGG |
| ***PNC1-E*** | GACCACTGTCCTGCTGGATT | TTGTGGGCCTTCAACTCTTC |
| ***NTH1*** | GAGTCGGGGTTTTTCTTTGAC | CGGATTCGTATGACGTTCTGT |
| ***ΝΑΤ4*** | TATATGAGGCGCTTGGGTTC | GTGACGAATTGTGGGTGATG |
